# The Legal Status of the Term “Reputable” as Applied to Dental Colleges

**Published:** 1902-08

**Authors:** Charles C. Chittenden

**Affiliations:** Madison, Wis.


### THE LEGAL STATUS OF THE TERM "REPUTABLE" AS APPLIED TO DENTAL COLLEGES.[1]

BY CHARLES C. CHITTENDEN, D.D.S., MADISON, WIS.

UNIFORMITY of dental laws and standards in the various States of this country has been a consummation long sought and most devoutly wished, by those men who have followed as well as those who have dictated and guided the development of our educational methods, with a view to placing the dental profession of this country where it rightfully belongs before the civilized world.

The comparative ease with which the legal right to establish and maintain schools and colleges in any State or Territory is secured is only exceeded by the great difficulties that have presented in all attempts to unify methods, curricula, and standards for the conferring of the dental degree.

[1] Read before the Section on Stomatology, American Medical Association, Saratoga Springs, June, 1902.

As schools increased in number, the estimated value of the dental diploma became more and more an unknown and uncertain quantity, which condition resulted in an effort towards the establishment by the people, through their legislative representatives, of some legal process of measuring and weighing the character and quality of any man's preparation and fitness before permitting him to offer his professional services to the public.

Since for obvious reasons a national standard could not be secured through Congress, it was left for each State to enact its own police regulations. Through the power of association which has been steadily growing during the past forty years, concerted action in the different States was possible in securing legislative enactment. A general and uniform draft of statute was prepared and recommended to the several States by committees selected for that purpose. But concert of action does not always bring uniformity of outcome.

The result has been a multiplicity and variety of dental laws almost as different in their construction as it is well possible to conceive—viewed superficially. For instance, forty-seven States and Territories of this country have enacted dental laws, in all but one of which a board of dental examiners has been created.

In twelve of these States no mention is made of the existence of a dental college. Any and all persons so desiring may be examined as to their fitness (no matter where acquired) to practise, and the board in its discretion licenses or rejects them.

Eight other States, on the other hand, will admit no one to examination who is not a graduate of a reputable dental college.

One State, Wyoming, has no board, but admits no one to practice without registering a diploma from a reputable college.

Added to these nine are twenty-six other States which, while admitting all who may so wish to examination by the board, at the same time direct the board to grant license without examination to those persons possessing a diploma from a dental college which complies with such standards and requirements as the law provides.

It therefore transpires that of the forty-seven enactments in this country for the regulation of the practice of dentistry, thirty-five specifically mention the possession of a diploma from a recognized or reputable dental college as a necessary or, at least, an important factor in securing a license to practise dentistry.

The different State laws make different specific requirements of colleges whose diplomas they accept as a qualification for license, but practically all laws provide that the college, after fulfilling all other specifications, shall, in addition thereto, be "reputable." In each and every case the board of examiners is left without any instruction as to the method of procedure in determining the reputability of a college, or as to the scope and meaning of the word "reputable." It is simply prescribed that the college must be "reputable," thus placing upon the board the judicial duty of establishing criteria of reputability and choosing its own methods of applying them to colleges when called to act officially.

Naturally, when standards have been fixed by a board which conflicted with the interests, financial or otherwise, of a certain school, and measured by which that school's diplomas are discredited, a definition of the board's powers and limitations has been sought by the use of mandamus proceedings. And there are several decisions extant on the statute-books of different States, all sustaining the position of the boards where there was no question as to the competence and good faith of the examiners.

It remained, however, for the Supreme Court of Wisconsin, in a decision handed down January 7, 1902, in the mandamus suit of W. L. Coffey *vs.* State Board of Dental Examiners, to so fully define and elucidate the whole question of the Board's powers, and of what the statute means by the word "reputable," as to set at rest any further cause or excuse for mandamus proceedings.

The State board had officially refused to accept Coffey's diploma in lieu of examination for license by the adoption of the following resolution:

WHEREAS, After due and careful consideration, acting under and pursuant to the provisions of Chapter No. 56 c. of the Wisconsin Statutes, this board has decided that in the opinion and according to the best judgment of the board, "the Department of Dental Surgery, ——— College of ———," is not a reputable dental college; therefore,

*Resolved,* That this board does hereby refuse to accept the diplomas of said Department of Dental Surgery, ——— College of ———, in lieu of examination for license to practise dentistry in this State.

Under mandamus proceedings in the lower court, all the reasons why the board declared this certain college to be, in its judgment, "not reputable" were brought out on the witness stand. These included, besides proof of certain misrepresentations by members of the faculty, under official questioning by the board as to facts concerning the school and its management, deviation from generally accepted standards in matriculation and transfer of students, in equipment, in curriculum, in methods of and facilities for instruction and training, and in several other particulars as to the methods of conducting the school.

The lower court held that the board had erred in some of its proceedings, and declared the school "reputable."

The Board appealed from this decision to the highest tribunal in the State.

The Supreme Court reversed the decision of the lower court and sustained the action of the State board at every point in a most exhaustive opinion reviewing the testimony brought out in the lower court.

The following is the summing up of the law points by the Court:

STATE OF WISCONSIN—IN SUPREME COURT.

STATE OF WISCONSIN ex. rel. W. L. COFFEY, Respondent,
*vs.*
C. C. CHITTENDEN et al., constituting the State Board of Dental Examiners, Appellants.

SYLLABUS.

1. The scope of mandamus proceedings to coerce a person or board to the performance of a judicial or quasi-judicial duty extends only to compelling such person or board to act, not to directing him or it how to act, unless the underlying facts are undisputed, leaving no reasonable ground for action other than one way.

2. If a person or board is clothed with judicial or quasi-judicial power in the determination of questions of fact and the taking of some specific action upon such determination, and fails to make a proper investigation of such questions, it is not within the function of a mandamus proceeding, predicated on such neglect, for the court to assume and exercise the duty of such person or board and make such investigation.

3. The character of a dental college or other institution of learning at a particular time may be established by evidence of its character at a prior time not so remote but that it would be reasonable to assume that the prior condition still exists. The rule applies that, "when the existence of a person, a personal relation, or a state of things is once established by proof, the law presumes that the person, relation, or state of things continues to exist as before, until the contrary is shown, or until a different presumption is raised, from the nature of the subject in question."

4. The character of a dental college in April of one year is evidentiary of its character in May of the next year, and may have sufficient probative force in that regard to reasonably establish such later character.

5. The word "reputable," as applied to dental colleges in the law authorizing the State Board of Dental Examiners to license persons to practise the profession of dentistry, without examination, who have graduated at such college having certain specified requisites, means *reputable* in the general sense in which the term is ordinarily used,—worthy of repute or distinction, held in esteem, honorable, praiseworthy.

6. In passing upon the application of a graduate of a dental college for a license to practise his profession in this State, the Board of Dental Examiners must determine whether his diploma comes from a reputable source as an independent fact, considering the term "reputable" in its ordinary sense and measuring the character of the college from the standpoint of men competent to judge thereof by reason of their scientific attainments in the line of work for which such a college stands.

7. The State Board of Dental Examiners, proceeding reasonably, is the sole tribunal under the statutes to determine the questions of fact to be solved, precedent to the licensing of a person to practise the profession of dentistry in this State.

8. When a graduate of a dental college applies to the State Board of Dental Examiners for a license to practise his profession, the burden of proof is upon him to establish the reputability of such college.

9. The State Board of Dental Examiners, having once determined the character of a dental college, may properly consider all questions in regard thereto at rest till, by lapse of time or otherwise, some reasonable ground exists for believing that its character may probably have changed.

10. The State Board of Dental Examiners having once determined the character of a dental college, within all reasonable limits, when and under what circumstances the subject shall be re-examined rests solely in its discretion.

11. Since the law does not define the method by which the State Board of Dental Examiners shall proceed to determine the reputability of a dental college when that is material to its official action, such board may perform its duty in that regard in any reasonable way it may deem proper, and candidates for licenses to practise the profession of dentistry must submit to its judgment so long as they are within the boundaries of reason and common sense.—Marshall, J.

Beyond a peradventure the foregoing syllabus definitely settles several things heretofore misunderstood or left in doubt.

*First.* The intention and meaning of the word "reputable" as applied to dental colleges is "worthy of repute or distinction, held in esteem, honorable, praiseworthy."

*Second.* The question of reputability of a college as an independent fact is left to the decision of "men competent to judge thereof by reason of their scientific attainments in the line of work for which such a college stands."

*Third.* The State board, proceeding reasonably, is the sole tribunal under the statutes to determine the questions of fact to be solved.

*Fourth.* The burden of proof is upon the graduate to establish the reputability of his college.

*Fifth.* The State board having once determined the character of a dental college within all reasonable limits, when and under what circumstances the subject shall be re-examined rests solely in its discretion.

*Sixth.* The board may perform its official duty of determining the reputability of a dental college in any reasonable way it may deem proper, and candidates for licenses to practise the profession of dentistry must submit to its judgment so long as they are "within the boundaries of reason and common sense."

The deduction of your essayist from all this is, that the question of unification of dental laws and interstate reciprocity of license, together with unification of dental educational standards on any reasonably high plane "within the bounds of common sense," is in the hands and entirely within the province and power of the State boards, provided they will act in concert in carrying out the high duties imposed upon them as the dental police officers of their several States, and this, too, without the enactment of any new dental legislation whatever.

The National Association of Dental Examiners and the National Association of Dental Faculties have enacted and adopted rules and educational standards that are acceptable to the profession at large, and have arrived at what general standards should be fixed as the criterion of reputability, but in order to make these "reasonable" standards nationally uniform it will be imperative that each and every State board in the Union adapt them to its use under its State laws and adopt them as its own standard of reputability.

Surely a standard built on such a broad foundation must be considered as coming "within the boundaries of reason and common sense," and therefore may be applied freely in judging the value of the diplomas of any applicant for license in any State.

In the twelve States first mentioned, where the law compels the examination of everybody and does not make any mention of a diploma (taking the State of Massachusetts as an example), the character and scope of the examination is left absolutely to the discretion and judgment of the board; therefore it is entirely competent for that board to make the possession of a diploma from a "reputable" college as prominent a factor in the examination of a postulant for license as they may deem wise, and thus enables the boards of these twelve States to have as much to say as to what constitutes a reputable dental college as those of the remaining thirty-five States and Territories where statutes do give a specified value to the diploma of a reputable college.

So it will be seen that the statute in every State in the Union grants its State board all the judicial power necessary to join in the establishing of absolute national standards of dental educational requirements.

There is absolutely nothing in all this proposed unification of standards, etc., by the boards that can react in any but a beneficial manner upon the schools themselves.

The element of commercial competition will be eliminated where it exists, and in its stead the spirit of emulation will be greatly stimulated.

The improvement and advancement in the general character of the dental college course in the past very few years is a fact which gives great promise for the future of dental education in this country.

Few can know and appreciate at how great an expense of energy, time, and strength, and with what sacrifice of self and the comforts of life, the honest dental teacher carries on his labor of love. Many of our brightest and best men have shattered their health and shortened or sacrificed their lives for the advancement and dissemination of knowledge in our colleges, and in almost no instance has the pecuniary return been in any sense adequate to the sacrifices made, and still the signs of the times indicate that the end is not yet.

The schools are building themselves on broader educational foundations, fitted to support a more elaborate and symmetrical superstructure, whose power and beauty shall command the respect and admiration of the civilized world, as well as set the pace for all Christendom.

School men have at last come to understand that the examiners, with the law at their backs, are a tower of strength in support of all honest educational efforts, as well as a menace to all forms of pretence and sham.

The only way in which this country can establish and maintain its supremacy in dental educational standards over the rest of the world, is by a concerted national legal standard being made by the legal guardians on whom the sacred trust is imposed—and these guardians must deserve and command the confidence and co-operation of the colleges, as well as the profession at large.

The question confronting us to-day is simply whether we as a profession will arise and enter into our birthright.

